# Supplementary material for: Simultaneous selection of nanobodies for accessible epitopes on immune cells in the tumor microenvironment
Source: Nat Commun. 2023 Nov 17;14:7473. doi: 10.1038/s41467-023-43038-z (PMC10656474; doi:10.1038/s41467-023-43038-z)
Supplement: Supplementary file 3 — Reporting Summary [file 41467_2023_43038_MOESM3_ESM.pdf]

nature portfolio

Corresponding author(s): Todd A Aguilera

Last updated by author(s): 10-1-2023

Reporting Summary

Nature Portfolio wishes to improve the reproducibility of the work that we publish. This form provides structure for consistency and transparency in reporting. For further information on Nature Portfolio policies, see our Editorial Policies and the Editorial Policy Checklist.

Statistics

For all statistical analyses, confirm that the following items are present in the figure legend, table legend, main text, or Methods section.

n/a	Confirmed	
☐	☒	The exact sample size (*n*) for each experimental group/condition, given as a discrete number and unit of measurement
☐	☒	A statement on whether measurements were taken from distinct samples or whether the same sample was measured repeatedly
☐	☒	The statistical test(s) used AND whether they are one- or two-sided<br>*Only common tests should be described solely by name; describe more complex techniques in the Methods section.*
☐	☒	A description of all covariates tested
☐	☒	A description of any assumptions or corrections, such as tests of normality and adjustment for multiple comparisons
☐	☒	A full description of the statistical parameters including central tendency (e.g. means) or other basic estimates (e.g. regression coefficient) AND variation (e.g. standard deviation) or associated estimates of uncertainty (e.g. confidence intervals)
☐	☒	For null hypothesis testing, the test statistic (e.g. *F*, *t*, *r*) with confidence intervals, effect sizes, degrees of freedom and *P* value noted<br>*Give P values as exact values whenever suitable.*
☒	☐	For Bayesian analysis, information on the choice of priors and Markov chain Monte Carlo settings
☒	☐	For hierarchical and complex designs, identification of the appropriate level for tests and full reporting of outcomes
☒	☐	Estimates of effect sizes (e.g. Cohen's *d*, Pearson's *r*), indicating how they were calculated

Our web collection on statistics for biologists contains articles on many of the points above.

Software and code

Policy information about availability of computer code

Data collection	NanoDropTM2000 (Thermo Fisher Scientific), 2100 Bioanalyzer (Agilent), UVP ChemStudio PLUS,<br>Sanger sequencing (core service), NGS sequencing (Illumine-MiSeq) (core service),<br>Orbitrap Elite (Thermo Fisher Scientific) (core service),<br>TC20 automated cell counter (BioRad),<br>Flow Cytometry: FACSDiva v9.0.1
Data analysis	NGS data analysis: MiXCR V3, IMGT Alpaca IG reference library, VDJtools, Immunarch,<br>Protein Discoverer 2.4<br>Single cell sequencing analysis: STAR version 2.7.2a, Seurat (3.2.3), R (4.0.3), topGO version 2.42.0, SingleR and celldex with ImmGenData, org.Mm.eg.db version 3.12.0, dittoseq version 1.2.5, SCP version 0.2.6<br>Confocal microscopy: NIS-Elements Version (5.4.1), Fiji ImageJ version 2.3.05 with Plugin JaCOP version 2.1.4. Microsoft Excel (Microsoft Office Professional Plus 2016),<br>Modeling: RosettaSuite version 3.13,<br>Flow Cytometry: FlowJo v10.9

For manuscripts utilizing custom algorithms or software that are central to the research but not yet described in published literature, software must be made available to editors and reviewers. We strongly encourage code deposition in a community repository (e.g. GitHub). See the Nature Portfolio guidelines for submitting code & software for further information.

Data

Policy information about availability of data

All manuscripts must include a data availability statement. This statement should provide the following information, where applicable:

- Accession codes, unique identifiers, or web links for publicly available datasets
- A description of any restrictions on data availability
- For clinical datasets or third party data, please ensure that the statement adheres to our policy

Data availability
Materials, protocols, and data are available upon request. Some requests may be subject to materials transfer agreement (MTA). Lead Contact: Todd.Aguilera@utsouthwestern.edu.
Materials availability: Some phage libraries may not be available unless acceptable negotiated MTA. Specific sequences and enrichment information will not be available until appropriate subsequent validation and protections are secured. All other materials and protocols are available upon request.
Data: All data can be made available and phage sequence data can be available subject to a negotiated MTA or data use agreement. Single cell RNA sequencing data has been deposited at 10.5281/zenodo.7557410. Mass Spectrometry data for Nb1 target identification has been deposited on MassIVE, accession number # MSV000092458
URL: https://massive.ucsd.edu/ProteoSAFe/dataset.jsp?task=5404e07d961648b79b5ffb528136cf05. Source data are provided as a Source Data file.

Code availability
No custom code was used to generate or process the data described in the manuscript.

Research involving human participants, their data, or biological material

Policy information about studies with human participants or human data. See also policy information about sex, gender (identity/presentation), and sexual orientation and race, ethnicity and racism.

Reporting on sex and gender	NA
Reporting on race, ethnicity, or other socially relevant groupings	NA
Population characteristics	NA
Recruitment	NA
Ethics oversight	NA

Note that full information on the approval of the study protocol must also be provided in the manuscript.

Field-specific reporting

Please select the one below that is the best fit for your research. If you are not sure, read the appropriate sections before making your selection.

[x] Life sciences [ ] Behavioural & social sciences [ ] Ecological, evolutionary & environmental sciences

For a reference copy of the document with all sections, see nature.com/documents/nr-reporting-summary-flat.pdf

Life sciences study design

All studies must disclose on these points even when the disclosure is negative.

Sample size	For each round of biopanning and selection there were three mice for each group used for each round or analysis unless otherwise stated in the manuscript. Sample sizes were determined for biologic replicates but not for a formalized statistical comparison only for biologic enrichment capture from the in vivo biopanning.
Data exclusions	There were no data exclusions. There were additional sample groups for the sorting and scRNAsequencing results described in figure. These data will be used for upcoming publication.
Replication	Each experimental setup is described in the text regarding the replication. For all mouse biopanning experiments at least three independent mice were used for selection. For validation or discovery experiments like the immunopreciptation, we performed 3 independent pull downs and LC/MS/MS experiments to corroborate PHB2 as the binding target. There were multiple confocal experiments for the Nb1 immunofluorescnece imaging in addition to measurement of multiple cells in each experiment and treatment group.
Randomization	For each animal experiment there was a cohort of animals prepared with tumors. They were then randomized to receive the identified phage injection without any other biologic selection criteria besides verifying that the tumor sizes were in the appropriate range.
Blinding	There was no blinding to group allocation in this study.

Reporting for specific materials, systems and methods

We require information from authors about some types of materials, experimental systems and methods used in many studies. Here, indicate whether each material, system or method listed is relevant to your study. If you are not sure if a list item applies to your research, read the appropriate section before selecting a response.

Materials & experimental systems

n/a	Involved in the study
	☒ Antibodies
	☒ Eukaryotic cell lines
☒	Palaeontology and archaeology
	☒ Animals and other organisms
☒	Clinical data
☒	Dual use research of concern
☒	Plants

Methods

n/a	Involved in the study
☒	ChIP-seq
	☒ Flow cytometry
☒	MRI-based neuroimaging

Antibodies

Antibodies used

Antigen (Clone / Fluorochrome / Catalog Number / Vendor)
CD45 (30-F11 / BV421 / 103134 / Biolegend)
CD11b (M1/70 / BV605 / 101257 / Biolegend)
CD11c (N418 / AF488 / 117311 / Biolegend)
CD11c (N418 / PE/Cy7 / 117318 / Biolegend)
CD8a (53-6.7 / BV510 / 100752 / Biolegend)
CD4 (GK1.5 / BV785 / 100453 / Biolegend)
CD25 (PC61 / PE / 102008 / Biolegend)
CD25 (PC61 / PE/Cy5 / 102010 / Biolegend)
CD19 (6D5 / PE/Cy7 / 115520 / Biolegend)
I-A/I-E (M5/114.15.2 / PerCP/Cy5.5 / 107626 / Biolegend)
His-Tag (polyclonal / - / 2365S / Cell Signaling technology)
His-Tag (J095G46 / APC / 362605 / Biolegend)
PHB2 (1D9C7 / - / 50 173 6851 / Thermo Fisher Scientific)
VHH (96A3F5 / - / A01860 / GenScript)
Mouse IgG (polyclonal / AF488 / A11029 / Thermo Fisher Scientific)
Rabbit IgG (polyclonal / APC / A10931 / Thermo Fisher Scientific)

Validation

All antibodies were validated by the manufacturers (Biolegend, Cell Signaling Technology, Thermo fisher Scientific, and GenScript). More details can be found on their respective websites.

Eukaryotic cell lines

Policy information about cell lines and Sex and Gender in Research

Cell line source(s)

Py8119 (also available from ATCC PY8119 CRL-3278) and Py117 breast cancer cell lines from female mice were derived from spontaneous tumors of transgenic MMTV-PyMT mice congenic in the CL57BL/6 background, provided by Ellies lab. MC38 (also available at Kerafast MC-38 ENH204-FP) is a colon cancer line from a female mouse and was provided by Engleman lab at an early passage. H1299 (also available from ATCC NCI-H1299 CRL-5803) is a human non-cell lung carcinoma derived from white male and provided by Story lab at an early passage.

Authentication

Py8119 and Py117 were authenticated by morphology after reception from Ellies lab. They have been analyzed by STR profiling and not identified as another cell line. MC38 and H1299 were authenticated by STR profiling (IDEXX BioAnalytics).

Mycoplasma contamination

All cell lines were tested negative for mycoplasma contamination using ATCC PCR Mycoplasma Detection Kit (Manassas, VA).

Commonly misidentified lines
(See ICLAC register)

No commonly misidentified lines were used.

Animals and other research organisms

Policy information about studies involving animals; ARRIVE guidelines recommended for reporting animal research, and Sex and Gender in Research

Laboratory animals

C57BL/6 wild-type mice with age groups ranging from 6 to 8 weeks, obtained from Jackson Laboratories (Bar Harbor, ME). Mice were housed with a standard day dark/light cycle 6:00 am to 5:59 pm, housed at ambient temperature, and humidified ventilated air.

Wild animals	Study did not involve wild animals.
Reporting on sex	All female mice were used for breast cancer tumor models given the sex of the donor cells and predominance of approximately 99% breast cancer arising in women
Field-collected samples	Study did not involve samples collected from the field.
Ethics oversight	All animal procedures were conducted according to the NIH guidelines for the care and use of laboratory animals and biological safety. Animal protocols were approved by the University of Texas Southwestern Medical Center Institutional Animal Care and use Committee (IACUC) under protocol number 102240 and the facility is AAALAC accredited.

Note that full information on the approval of the study protocol must also be provided in the manuscript.

Flow Cytometry

Plots

Confirm that:

- [x] The axis labels state the marker and fluorochrome used (e.g. CD4-FITC).
- [x] The axis scales are clearly visible. Include numbers along axes only for bottom left plot of group (a 'group' is an analysis of identical markers).
- [x] All plots are contour plots with outliers or pseudocolor plots.
- [x] A numerical value for number of cells or percentage (with statistics) is provided.

Methodology

Sample preparation	Tumor, draining lymph nodes, and spleen from Py8119 or Py117 tumor-bearing mice were excised and dissociated into a single cell suspension. Spleen and lymph nodes were processed by gentle homogenization and 40 µm filtration in 10% serum-supplemented medium. Tumor tissue samples were digested with 4 ml of serum-free medium containing 5 µM liberase and 100 µM DNAse for 40 min at 37 °C with shaking at 70 RPM. The tumor digestion protocol #4 was performed by using the gentleMACS Octo tissue dissociator (Miltenyi Biotec) with three 10 min rounds of processing over 40 min. Serum-supplemented medium was added to the digestion and cells were filtered through a 40 µm filter. The cell suspension was pelleted, then RBCs were lysed for 5 min at 4 °C in ACK buffer, then cells were resuspended in fresh medium. Lymphocytes were isolated via ficoll gradient, suspended in fresh medium, and washed. Cells were resuspended in MACS staining buffer and passed through LS columns with dead cell removal magnetic bead kit (Miltenyi Biotec). Cells were then suspended in PBS supplemented with 5 % BSA.<br>For confirmation of magnetically enriched cells, two million cells were mixed with 3.0 µl of Fc block and 0.25 µl of Zombie NIR in a volume of 100 µl MACS staining buffer + 5 % BSA, incubated at 4 °C for 15 min, and washed once with staining buffer. Samples are then incubated with antibodies at 4 °C for 30 min in darkness. Cells are washed once with buffer and resuspended in 300 µl staining buffer.<br>For nanobody binding experiments, two million cells were mixed with 3.0 µl of Fc block and 0.25 µl of Zombie NIR, incubated at 4 °C for 15 min, and washed once with PBS. For His labeled nanobody alone, protein (2 to 4 µg) was added to the Fc block-treated cells with a final reaction volume of 100 µl and incubated for 30 min at 4 °C. For nanobody binding experiments, cells were washed once with PBS, then APC-conjugated His-tag antibody was added, and the mixture was incubated further for 15 min at 4 °C. As a negative control, APC-conjugated His-tag antibody was added to the cells treated with no nanobody proteins. For Venus fusion proteins 2-4 ug were incubated with no secondary antibodies to then detect protein directly using venus fluorescence. Cells were washed twice and incubated with antibodies for immune cell identification at 4 °C for 30 min in darkness. After washing once, cells were suspended in PBS before flow cytometry analysis.
Instrument	For analysis the LSR Fortessa (BD Biosciences) was used, for sorting of cells for single cell sequencing experiments Aria II flow sorter (BD Biosciences) was used.
Software	For collection: BD FACSDiva v9.0.1, for analysis: FlowJo™ v10.9.
Cell population abundance	Magnetically sorted cell abundance was assessed using flow cytometry. CD11b positively selected cells Cells are 57 % positive for CD11b, CD11c positively selected cells are 66 % positive for CD11c. CD8 negatively selected cells have 99% purity. CD4+CD25- negatively selected cells are 55 % pure and CD4+CD25+ positively selected cells are 35 % pure.
Gating strategy	For magnetically sorted cells, gating is indicated in Supplementary Figure 2. Single cell, live lymphocytes were identified and debris was excluded using FSC/SSC gates. Then CD45+ cells were separated by MHC Class II negative/positive gates for subsequent identification of CD8+/CD4+/CD25+ or CD11c+/CD11b+ populations respectively. For identification of positive CD25, CD11c or CD11b gates, FMO for the respective marker were used. For nanobody binding, gating is indicated in Fig 5. In short, FSC/SSC identified single cell lymphocytes were gated for CD45+ cells and then subsequently for CD11c+CD11b, CD11c-CD11b+, CD8+, and CD4+ cells.

- [x] Tick this box to confirm that a figure exemplifying the gating strategy is provided in the Supplementary Information.
