# Supplementary figures and images for: Simultaneous selection of nanobodies for accessible epitopes on immune cells in the tumor microenvironment
Source: Nat Commun. 2023 Nov 17;14:7473. doi: 10.1038/s41467-023-43038-z (PMC10656474; doi:10.1038/s41467-023-43038-z)

# Purified Venus-Nanobody Fluorescent Gels

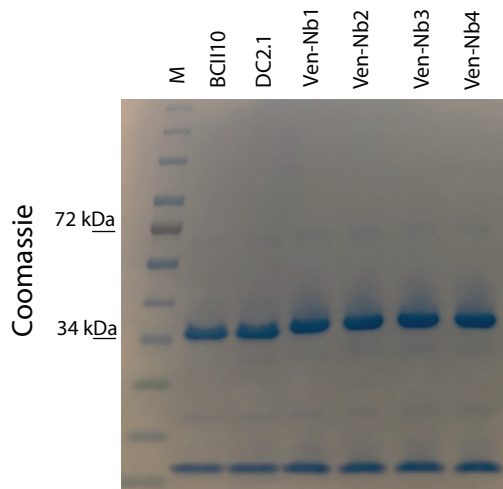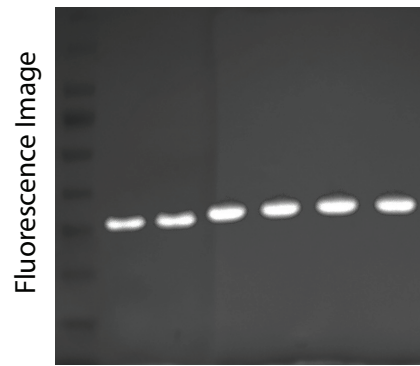

## Purified Venus-Nanobody aHis Western

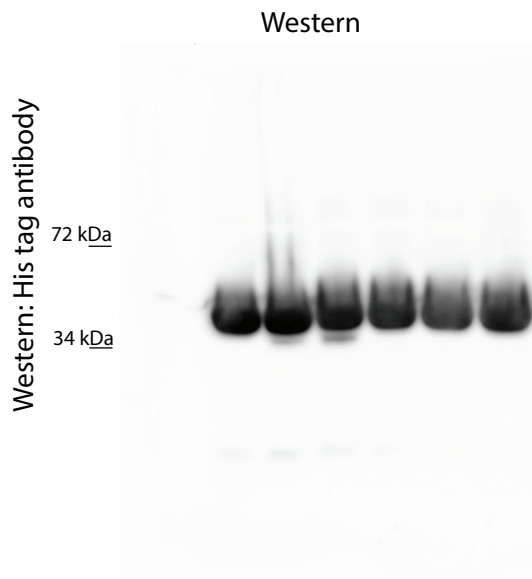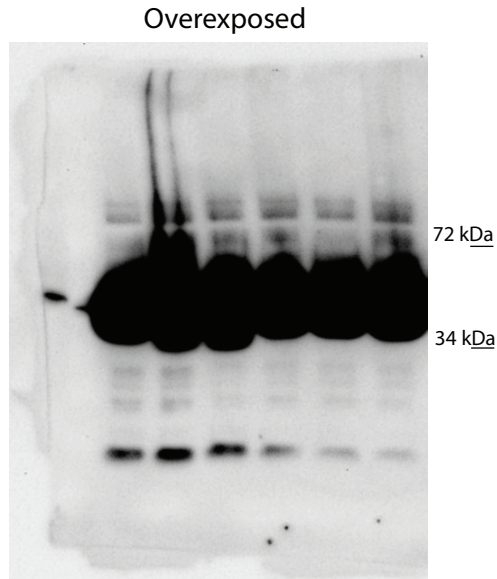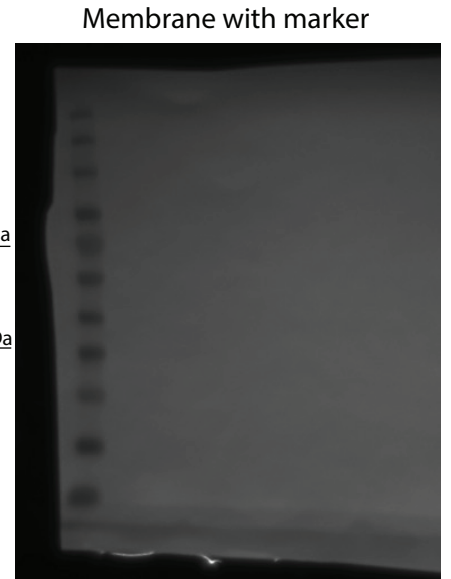

Supplement: Supplementary file 4 — Source Data [file 41467_2023_43038_MOESM4_ESM.zip › Fig7B_Source_Data.pdf]
